# Medication adherence in the curricula of future European physicians, pharmacists and nurses – a cross-sectional survey

**DOI:** 10.1186/s12909-025-06909-1

**Published:** 2025-03-05

**Authors:** Hanna Gottlieb, Laura Seghers, Francisca Leiva-Fernandez, Cristina Mihaela Ghiciuc, Gaye Hafez, Maria Teresa Herdeiro, Ana Tomas Petrović, Teddy Novais, Marie P. Schneider, Alexandra Dima, Marie Ekenberg, Björn Wettermark, European Network to Advance Best Practices Technology on Medication Adherence (ENABLE)

**Affiliations:** 1 Department of Pharmacy, Faculty of Pharmacy, Uppsala University, Box 580, Uppsala, 751 23 Sweden; 2 Department of Pharmaceutical and Pharmacological Sciences, KU Leuven, Leuven, Belgium; 3 Andalusian Health Service, Malaga Biomedical Research Institute (IBIMA-Plataforma BIONAND), University of Malaga, Malaga, Spain; 4 Clinical Pharmacology and Algesiology, Faculty of Medicine, Grigore T, Popa University of Medicine and Pharmacy, Iasi, Romania; 5 Department of Pharmacology, Faculty of Pharmacy, Altinbas University, Istanbul, Turkey; 6 Institute of Biomedicine (iBiMED), Medical Sciences Department, University of Aveiro, Aveiro, Portugal; 7 Department of Pharmacology, Toxicology and Clinical Pharmacology, Faculty of Medicine, University of Novi Sad, Novi Sad, Serbia; 8 Department of Pharmacy, Pharmaceutical Unit, Charpennes Hospital, Hospices Civils de Lyon, University Hospital of Lyon, Villeurbanne, 69100 France; 9 Lyon Institute for Aging, Hospices Civils de Lyon, Lyon, 69000 France; 10 Research on Healthcare Performance (RESHAPE), INSERM U1290, University Lyon1, Lyon, 69000 France; 11 School of Pharmaceutical Sciences, University of Geneva, Geneva, Switzerland; 12 Institute of Pharmaceutical Sciences of Western Switzerland, University of Geneva, Geneva, Switzerland; 13 Avedis Donabedian Research Institute, Autonomous University of Barcelona, C / Provença 293, Barcelona, Spain; 14 Health Technology Assessment in Primary Care and Mental Health (PRISMA), Institut de Recerca Sant Joan de Déu, Santa Rosa 39-57, Esplugues de Llobregat, 08950 Spain; 15 Consortium “Centro de Investigación Biomédica en Red” Epidemiology and Public Health (CIBERESP), Madrid, Spain; 16 Pharmacy Center, Institute of Biomedical Science, Faculty of Medicine, Vilnius University, Vilnius, Lithuania

**Keywords:** Education, Medicine, Pharmacy, Nursing, Medication adherence

## Abstract

**Aim:**

Many patients are not taking their medicines. It has substantial negative medical and economic consequences for patients and healthcare systems but there is limited knowledge on how medication adherence is integrated in medical education. This study seeks to investigate to what extent students in medicine, pharmacy and nursing in Europe are taught about medication adherence.

**Methods:**

A cross-sectional online survey was distributed to 731 persons teaching relevant courses across 142 European universities between February and June 2024. The survey addressed definitions of adherence and The ABC Taxonomy; methods to support adherence, methods to identify and monitor non-adherence; consequences and outcomes of non-adherence, and methods applied in teaching. They were also asked to provide links to their curricula. Responses from quantitative questions were analyzed descriptively. Word frequency and qualitative thematic analysis was used for the curricula inventory and analysis of free-text answers, respectively.

**Results:**

In total, 212 participants from 114 universities in 34 countries completed the survey. Respondents agreed to similar level on the need to enhance medication adherence teaching, with 72% in pharmacy, 71% medical, and 59% agreement in nursing education. The most taught topic across educations was the clinical impact of non-adherence, according to 89% in pharmacy, 84% medical, and 76% in nursing education. The ABC Taxonomy was taught in more than half of all pharmacy (73%), nursing (60%) and medical education (52%). In the qualitative analysis of free text-answers respondents emphasized the value of early, mixed method teaching. They reported a lack of guidance in teaching medication adherence, causing inconsistency in the educational quality and depth. Time constraints were highlighted as a significant challenge, while interprofessional collaboration and use of medication adherence technologies were seen as opportunities, though not widely implemented in teaching. The curricula inventory showed a substantial variance in how medication adherence content was described.

**Conclusion:**

There is a lack of consistent teaching on medication adherence in Europe, underlining the necessity to establish a unified curriculum incorporating the ABC taxonomy, and to include a more patient-centred approach to support medication adherence.

**Supplementary Information:**

The online version contains supplementary material available at 10.1186/s12909-025-06909-1.

## Background

Poor adherence to prescribed medicines, i.e., failure to follow recommended health behaviors and treatment advice, is a problem in the treatment of most chronic diseases [1]. Studies have found that less than half of all patients’ with long-term therapy take their medicines as indicated [2]. The consequences are considerable, with psychosocial and medical complications of disease, impaired quality of life and premature deaths as well as increasing health expenditure and waste of resources [1–3].

Five dimensions of determinants behind poor medication adherence have been identified including social and economic factors (e.g. socio-economic status, social support); health care team and system-related factors (e.g. accessibility of care, supply of medicines); condition-related factors (e.g., presence of symptoms, severity of disease); therapy-related factors (e.g. length of treatment, adverse effects) and patient-related factors (e.g., age, gender, cognitive ability) [2]. These dimensions embrace more than 750 individual factors related to non-adherence [4]. Access to medication is the first enabling step towards achieving the desired disease outcome but it becomes insufficient if the treatment plan is not successfully followed. Thus, medications adherence is as a prerequisite for high quality of care [2, 3]. Despite research and awareness-raising interventions for half a century as well as the integration of medication adherence as learning outcome in e.g. training in clinical pharmacology and therapeutics in Europe, medication non-adherence continues as a hurdle in the effective treatment of most medical conditions[1, 2, 5, 6].

Various interventions have been performed by health care providers (HCPs) with the ambition to improve medication adherence, mainly by targeting individual patient behaviors, however with limited success rates [5, 7, 8]. Most successful strategies includes improvement in communication between HCPs and patients which has shown to be important in increasing patient involvement in their medicines management [9–11]. A Cochrane review suggested that, counseling patients to enhance medication adherence should be a shared task between nurses, pharmacists and physicians [8]. Interprofessional collaboration, has also shown to be important in improving treatment outcomes and reducing health care expenditure [10, 12, 13]. However, most HCPs lack knowledge and awareness of the magnitude of non-adherence and its consequences, as well as the availability of efficient solutions for improvement [14, 15]. There is also limited knowledge on to what extent medication adherence is integrated in pre- and postgraduate education. Schneider & Aslani [16] investigated how medication adherence was integrated into the curriculum of pharmacy education across Europe and found that it is imbedded in courses within the pre- or postgraduate programs, often within a therapeutic area of study but that the teaching varied greatly across Europe [16]. In 2013, researchers from the UK suggested an educational framework with the intention of establishing a broad consensus statement for the education and training of HCPs on medication adherence [17]. In the same year, the EU-funded project, “Ascertaining Barriers for Compliance (ABC): policies for safe, effective and cost-effective use of medicines in Europe”, developed and distributed guidelines for the education of HCPs, with the aim of improving their skills in preventing and managing non-adherence [2]. A recent survey conducted by the EU COST ENABLE project, identified an expressed lack of training in adherence monitoring and assessment, methods for patient-empowerment and clear definitions of the HPC’s roles and tasks in medication adherence management [18]. With today’s available frameworks and recent extensive research on effective solutions to improve medication adherence, it is important to further understand how medication adherence is implemented in the curricula of medicine, pharmacy and nursing schools. Identifying knowledge gaps in education as well as good examples of practice could lay a basis for transforming these educations towards a more patient centred approach promoting safe and effective use of medicines. Therefore, this study seeks to investigate to what extent students in medicine, pharmacy and nursing in Europe are taught about medication adherence.

## Methods

### Study design

A mixed methods study was applied with a cross-sectional questionnaire distributed to European universities providing pre-graduate education of physicians, pharmacists and/or nurses. This was complemented by a qualitative content analysis of curricula provided by respondents upon request in the questionnaire.

### Setting and participants

The study covered schools of medicine, pharmacy and nursing at 142 European universities identified by searches on individual university websites, by the authors in their respective countries, and reaching out to persons within the European universities identified through scientific network of ENABLE COST Action, a scientific network including researchers active in medication adherence from 40 (89%) European countries [19].

The target group for the questionnaire was teachers responsible for relevant courses in medicine, pharmacy and nursing programs. Purposive and snowball sampling strategies were used in each university. The questionnaire was distributed by sending out emails to participants involved in courses that may include medication adherence, across Europe. For text analysis of curricula, official curricula were provided by some respondents through the questionnaire. The questionnaire was open from February to June 2024. The main respondents to the questionnaire were notified by e-mail and received a weekly reminder during three consecutive weeks.

### Questionnaire development and data collection

The survey structure was inspired by previous questionnaires on curricula and education evaluations in health care [20, 21]. Questions were formulated according to previous research on medication adherence and course evaluations [16, 17, 22, 23]. The questionnaire was subsequently evaluated by an expert group of 15 researchers active in the ENABLE COST action, and the survey was further tested for validity and reliability by seven teachers involved in teaching medication adherence within undergraduate pharmacy educations in different European countries. The questionnaire was reviewed using a think-aloud method [24] where all questions were addressed along with the introduction to the questionnaire; the time it took to complete; the legibility of the questions; the order and number of close-ended vs. open-ended; and any other comments. The survey involved Nominal scales, Likert scales, free-text questions and possibilities for free-text comments. It consisted of 44 questions divided into three sections. The respondents were also requested to submit the curricula of relevant courses, containing an element of medication adherence. The full questionnaire is available in supplementary material (see Additional file 1.).

#### Section 1. Characteristics and attitudes of the respondents

The first section of the questionnaire covered questions about respondents’ gender, professional role, level of education, experience in teaching and research in the field of medication adherence. The respondents were asked to provide the university and country that they were associated with. Furthermore, questions were asked about respondents’ attitude on the importance of medication adherence and respondents were asked to estimate the total number of hours devoted to medication adherence in their curriculum.

#### Section 2. Characteristics of the course(s)

In this section respondents were asked to provide specific information about the course(s) they taught/were involved in such as: the name of the course; the main target student groups(s) of the course; if it contained interprofessional collaboration; if there was any patient involvement; which teaching strategies (e.g. lectures, group discussions and clinical field studies), and materials (e.g. scientific articles, national/international guidelines and books) that were used. Respondents were also asked to attach the curriculum/syllabus of the course(s).

#### Section 3. Domains in medication adherence

The last section comprised four different domains identified within the topic of medication adherence that can be included in the teaching of HCPs. The domains were created to systematically categorize and define the questions within the survey and were defined based on scientific literature on medication adherence [1, 2, 16], course evaluations and feedback from the pilot study. The domains were: definitions of adherence and related terms; methods to identify and measure non-adherence; adherence monitoring and supporting methods in daily practice; and consequences and outcomes of non-adherence. Respondents were asked to specify which domains and subdomains that were included in the teaching of medication adherence. Furthermore, respondents were asked to specify teaching strategies, type of examination and if they included Medication Adherence Technologies (MATech) in their teaching. MATech are defined as evidence based devices, procedures or systems designed to support patients in their medication regimen (to initiate, implement and presist) [25]. Finally, free-text questions were included where respondents were asked to share opportunities/challenges in teaching medication adherence. The survey was conducted in English and was also translated to Spanish and French using a committee-based method [26]. A survey instrument was created electronically using the secure web-based software platform Research Electronic Data Capture (REDCap) and a REDCap program conducted by the Vanderbilt University [27, 28]. The data was collected and managed using the REDCap tool hosted at Uppsala University, Sweden. Map data and visualization was provided by Microsoft Bing Maps, with data provided by © GeoNames, © Microsoft, © Open Places, © OpenStreetMap and © TomTom. Word cloud was provided by WordItOut.

#### Data analysis

A completeness check was conducted after the questionnaires were submitted and only responses where the first section was completed were analyzed. Descriptive analysis on survey data, stratified by pharmacy-, medical-, and nursing program was conducted. Statistical analysis was performed using the chi-square test and the t-test for categorical- and continuous variables, respectively. Results were either presented in proportions with p-value or mean values ± 95% confidence interval (CI95%). All analysis was performed using Microsoft Excel.

The analysis of the free-text statements in the questionnaire was done using a simplified qualitative content analysis [29, 30]. First, preliminary categories were defined with a deductive approach, based on the content of the questionnaire. The free-text answers were then summarized in codes, which were assigned to different categories. Main overarching themes were defined. Additionally, the emerging themes were compared to one another to avoid overlapping or duplication of responses. Classification of free text answers was done by peer review processes by two persons (HG and LS). After each pair conducted categorization and coding of the free text answers a consensus meeting was held to finalize the grouping of terms.

The curricula analyses focused on identifying the learning objectives related to medication adherence and involved reading through the curricula and searching for keywords, which was conducted with a peer-review process, where identification and paring of synonym key words were conducted by each pair and later compared and agreed on in a consensus meeting.

## Results

**Fig. 1:**
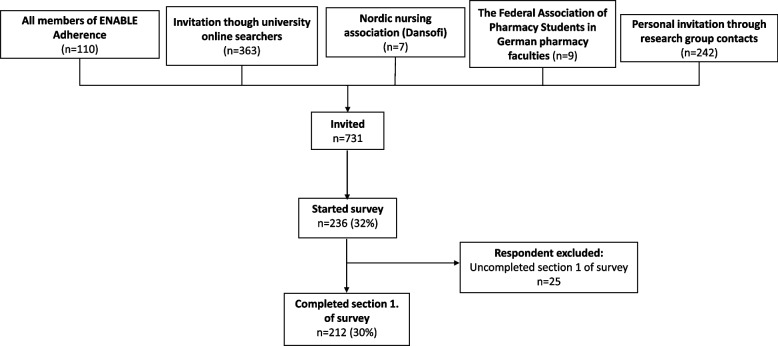
Flow chart of respondents

**Fig. 2:**
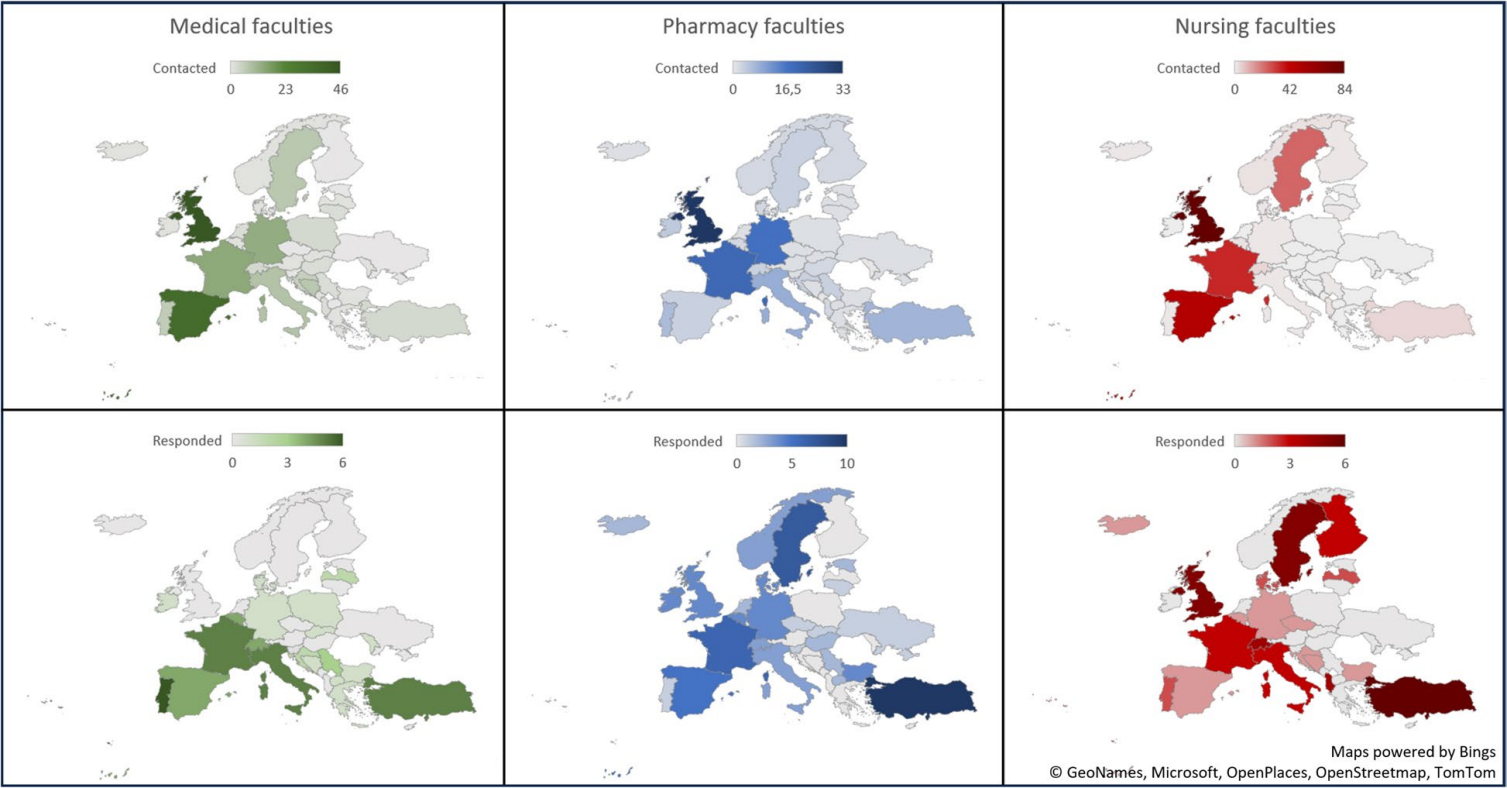
Participants geographic distribution for survey distribution and responses. Presented in numbers of faculties divided into medical-, pharmacy-, and nursing faculties.

The questionnaire was distributed to 731 persons, across 142 European universities, teaching or involved in the teaching of relevant courses (Fig. 1). Of whom, 212 participants from 114 universities in 34 countries completed the first section of the survey, resulting in a 30% response rate (Fig. 2). List of participating universities and countries is available in supplementary material (see Additional file 2.).

Pharmacy faculties responded to the highest degree (56%), while medical faculties and nursing faculties responded to a degree of 30% and 24%, respectively. Of all respondents, six reported teaching within all three faculties, four in pharmacy and medical school, two in pharmacy and nursing school, lastly eight in medical and nursing school.

### Participants’ demographic characteristics

Of the respondents, 63% were female, predominantly course leaders (45%) or teachers of a course (35%), with either a PhD as the highest education (34%) and/or a Full Professor title (24%) (Table 1). The years of teaching experience reported by the respondents varied, 27% reported having been teaching > 20 years and 20% for 6–10 years. Less than half responded that they were pursuing research (44%) or that they had previously pursued research in medication adherence (13%).

**Table 1:** Characteristics of respondents in survey on medication adherence in teaching

	**All** n (%)	**Pharmacy** n (%)	**Medical** n (%)	**Nursing** n (%)	**Missing** n (%)
**Total**	212(100)	82(38.7)	56(26.4)	58(27.4)	41(19.3)
**Sex & gender**					
Female	149(70.3)	66(79.3)	26(46.4)	42(72.4)	28(68.3)
Men	61(28.8)	17(8.0)	29(13.7)	15(7.1)	13(31.7)
Prefer not so say	2(0.9)	0(0.0)	1(0.5)	1(0.5)	0(0.0)
**Highest academic degree**					
BSc and MSc^a^	34(16.0)	7(8.5)	8(14.3)	19(32.8)	9(22.0)
PhD^b^	73(34.4)	35(42.7)	16(28.6)	22(37.9)	15(36.6)
Assistant/Associate Professor^c^	37(17.5)	18(22.0)	11(19.6)	8(13.8)	8(19.5)
Full Professor^c^	50(23.6)	21(25.6)	20(35.7)	9(15.5)	6(14.6)
Prefer not to say	6(2.8)	1(1.2)	1(1.79)	1(1.7)	3(7.3)
**Role**					
Course leader	95(44.8)	49(59.8)	25(44.6)	21(36.2)	19(46.3)
Teacher of a course	74(34.9)	27(32.9)	19(33.9)	28(48.3)	14(34.1)
Teacher not involved in the course	8(3.7)	2(1.2)	3(5.4)	4(6.9)	4(9.8)
Other	15(7.1)	4(4.9)	7(12.5)	4(6.9)	3(7.3)
Prefer not to say	3(1.4)	1(1.2)	2(3.6)	1(1.7)	1(2.4)
**Years of teaching experience**					
< 5 years	31(14.6)	11(13.4)	9(16.1)	11(19.0)	3(7.3)
6–10 years	43(20.3)	20	10(17.9)	13(22.4)	11(26.8)
11–15 years	38(17.9)	13(15.9)	11(19.6)	14(24.1)	6(14.6)
16–20 years	24(11.3)	13(15.9)	6(10.7)	5(8.6)	12(29.3)
> 20 years	58(27.4)	26(30.5)	18(32.1)	15(25.7)	9(22.0)
Prefer not to say	2(0.9)	0(0.0)	2(3.6)	0(0.0)	0(0.0)
**Pursuing research in MA**					
Not now, but previously	31(14.6)	13(15.9)	12(21.4)	6(10.3)	9(22.0)
Yes, I do	94(44.3)	55(65.9)	21(37.5)	19(32.8)	16(39.0)
No	67(31.6)	14(17.1)	21(37.5)	32(55.2)	3(0.0)
Prefer not to say	4(1.9)	1(1.2)	2(3.6)	1(1.7)	0(0.0)

In total, 20 subjects reported to be involved in two or three educations, hence there is an overlap in responses

^a^Including medical doctor and postgraduate certification in pharmacy

^b^Including HDR (Higher degree by research)

^c^Assistant professor (entry level position, building teaching/research portfolio); Associate professor (mid-career, tenured position); Full professor (senior, distinguished achievements in the field), MA (= medication adherence)

### Attitudes on the importance of medication adherence

**Fig. 3:**
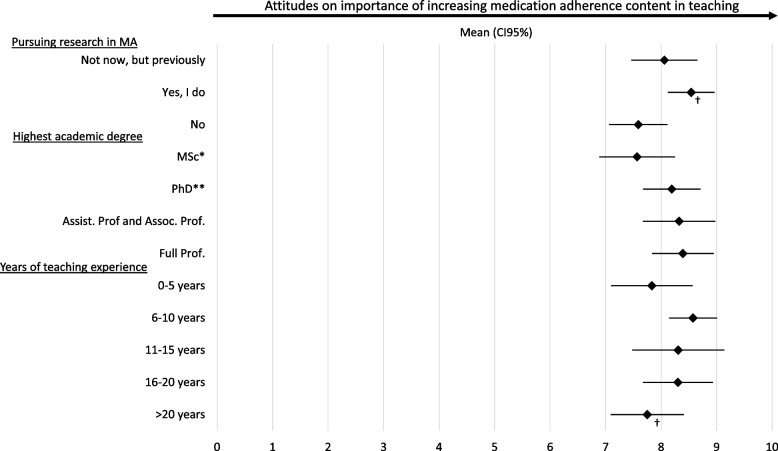
Attitude on the need to emphasize more attention on medication adherence in teaching. Expressed on a Likert scale (Strongly disagree (0) – Strongly agree (10)) presented in mean values and confidence interval 95% (CI95%), with subjects divided according to experience in research, highest academic degree and years of teaching experience. Independent t-test used to compare mean scores between groups (*p* < 0.05 indicates significance)

When asked if there is a need to emphasize medication adherence in the teaching of medical-, nursing- and pharmacy education on a scale of 0–10 (strongly disagree-strongly agree) the attitudes of the respondents were relatively similar with a mean value of 8.14 (confidence interval 95% (CI95%) 7.8 – 8. 4). Respondents in pharmacy (72%), medical (71%) and nursing education (59%) agreed to a similar degree that there is a need to emphasize teaching of medication adherence., rating above 8. Replies were stratified according to years of experience in teaching, highest education level, and research experience in medication adherence (Fig. 3). A significant difference (p = 0.005) was observed between respondents conducting research in medication adherence (mean: 8.5, CI95% 8.1 – 8.9) and respondents who have not performed any research in the topic (mean: 7.6, CI95% 7.1 – 8.1). A significant difference (p = 0.05) was also observed between subjects with 16–20 years of teaching experience (mean: 8.4 CI95% 7.7 – 8.9) and subjects teaching > 20 years (mean: 7.7, CI95% 7.1 – 8.4). No difference was observed between the different educational levels.

### Teaching on medication adherence

#### Domain 1. Definitions and terms

The definition of adherence was covered to a large degree amongst all three educations (pharmacy 82%; nursing 60%; medicine 52%) (Table 2). The ABC Taxonomy, providing a consensual and complete description of adherence to medicine, was included in more than half of all educations in pharmacy (73%), nursing (60%) and medicine (52%). Further, approximately 10% in medical and nursing education reported not including any definitions and terms in the teaching of medication adherence.

**Table 2:** Reported teaching content according to the different domains related to medication adherence in European universities

**Domain 1: Which of the definitions** **and terms are covered in the course(s)?**	**Pharmacy** (*n* = 82)	**Medical** (*n* = 56)	**Nursing** (*n* = 58)	**P-value**
The definition of adherence	67 (81.7)	38 (51.8)	35 (60.3)	< 0.05
The definition of adherence to medication	59 (72.8)	29 (52.0)	35 (60.3)	0.05
The definition of management of adherence	47 (58.0)	22 (39.3)	23 (39.7)	0.05
Adherence in relation to compliance	55 (67.1)	26 (46.4)	25 (43.1)	< 0.05
Adherence in relation to concordance	45 (55.6)	21 (37.5)	25 (43.1)	ns
The five determinants of adherence	55 (69.9)	28 (50.0)	24 (41.4)	< 0.05
None of them	0 (0.0)	5 (8.9)	6 (10.3)	< 0.05
**Domain 2: Which of the identifying and measurement methods are covered in the course(s)?**				
Patient reported: Talking with the patients, interviewing them, having a survey	61 (75.3)	41 (73.3)	29 (50.0)	< 0.05
Manual pill count and electronic detection of package entry	48 (59.3)	25 (44.6)	20 (34.5)	< 0.05
Direct measurements: drug or drug metabolite monitoring	48 (59.3)	27 (48.2)	18 (31.0)	< 0.05
Registries: Electronic prescription data, refill/dispensing data form pharmacies and electronic health records (EHRs)	57 (70.4)	25 (44.6)	16 (27.6)	< 0.05
Another method to measure medication adherence	12 (14.6)	5 (8.9)	4 (6.9)	ns
None of them	7 (8.6)	4 (7.1)	13 (22.4)	< 0.05
**Domain 3: Which of the monitoring and supporting medication adherence methods are covered in the course(s)?**				
Medication Management	61 (75.3)	37 (66.1)	29 (50.0)	< 0.05
Interprofessional collaboration and communication	47 (58.0)	21 (37.5)	24 (41.4)	< 0.05
Patient provider communication	54 (66.7)	31 (55.4)	35 (60.3)	ns
Patient education	56 (69.1)	35 (62.5)	40 (69.0)	ns
Patient engagement	47 (58.0)	22 (39.3)	24 (41.4)	ns
Behavioral change techniques	28 (34.6)	12 (21.4)	12 (20.7)	ns
Social Factors	29 (35.8)	13 (23.2)	16 (27.6)	ns
Digital tools	41 (50.6)	20 (35.7)	14 (24.1)	< 0.05
Another method to monitor or to support medication adherence	3 (3.7)	0 (0.0)	1 (1.7)	ns
None of them	3 (3.7)	4 (7.1)	4 (6.9)	ns
**Domain 4: Which of the consequences and outcomes of medication non-adherence are covered in the course(s)?**				
Economic impact	55 (67.1)	32 (57.1)	27 (46.5)	0.05
Clinical impact	72 (87.8)	47 (83.9)	44 (75.9)	ns
Social impact	39 (48.2)	22 (39.3)	18 (31.0)	ns
None of them	3 (3.7)	5 (8.9)	6 (10.4)	ns
**Do you teach about Medication Adherence Technologies (MATech) in the course?**				
Yes	38 (48.7)	10 (18.9)	12 (23.1)	< 0.05
No	35 (44.9)	37 (69.8)	38 (73.1)	< 0.05
Don't know	5 (6.4)	6 (11.3)	2 (3.8)	ns

#### Domain 2. Identifying and measurement methods

Compared to the other domains, identifying and measurement methods were included to the lowest extent, with 8.6% in pharmacy, 7.1% in medical and 22% in nursing education reporting to not include any measurement methods in the teaching of medication adherence (Table 2). Patient-reported methods, such as talking, interviewing and using surveys were reportedly the most taught methods in pharmacy (75%), medical (73%) and nursing education (50%). Registries, such as electronic prescription data and electronic health records were also included to a high extent in pharmacy education (70%), but not as common in medical (45%) and nursing education (28%).

#### Domain 3. Methods for monitoring and supporting medication adherence

Medication management, i.e., management of missed doses, a pill chart, management of side effects and interactions etc., was the most common method included in pharmacy (75%) and medical (66%) education. In nursing, patient education was the main included method for supporting medication adherence (69%). Interprofessional collaboration and communication were included in the pharmacy (58%), medical (38%) and nursing education (41%) to different extents. None of the listed methods for monitoring and supporting medication adherence were included in the teaching of 3.7% pharmacy, 7.1% medical, and 6.9% nursing education.

#### Domain 4. Consequences and outcomes of medication non-adherence

Amongst all subjects within medication adherence, the clinical impact of medication non-adherence was covered to the highest degree in pharmacy (88%), medical (84) and nursing education (76%). The social impact of medication non-adherence was included to the lowest degree with 48% pharmacy, 39% medical and 31% in nursing education. Further, 3.7% in pharmacy, 8.9% in medicine, and 10.4% in nursing reported not including the consequences and outcomes of medication non-adherence in teaching.

#### Medication Adherence Technologies (MATech)

Most respondents in pharmacy (45%), medical (70%) and nursing education (73%) reported that they do not include MATech in their teaching. Pharmacy education reported the highest use of MATech in teaching where half of all courses (49%) included it.

Presented in counts and percentage (%). The chi-square test was used to assess the difference between the three groups (p < 0.05 indicates statistical significance).

### Teaching methods and materials

**Fig. 4:**
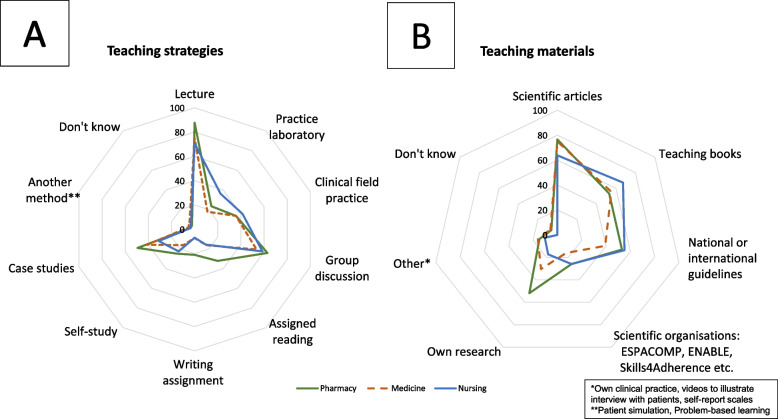
Reported use of teaching strategies (**A**) and materials (**B**) expressed in percentage (%) for pharmacy, medical and nursing education in European universities

Scientific articles were reported as the predominantly used teaching material in both pharmacy (76%), medical (75%) and nursing education (64%), followed by teaching books (pharmacy 53%; medical 55% and nursing 67%) (Fig. 4). The use of own research in teaching was used significantly more in pharmacy education (52%, p < 0.05), compared to medical (30%) and nursing education (17%).

Lectures were the dominant teaching strategy (pharmacy 88%; medical 75% and nursing 71%), followed by group discussions (pharmacy education 63%; nursing 59%; medical education 54%). Patients were included significantly more in pharmacy education (32%, p < 0.05). The largest proportion of pharmacy school respondents (37%) reported a total of 2–5 days dedicated to medication adherence. In contrast, 31% of nursing school respondents indicated that between half a day and one full day was devoted to medication adherence. Finally, 37% of medical school respondents reported 1–2 h allocated to this topic.

Written exams were reported as the most common examination type in pharmacy (62%), medical (60%) and nursing education (48%), followed by oral exams used in pharmacy (44%) medical (23%) and nursing education (21%). Other examination types reported by the respondents were objective structured clinical examination (OSCE), seminars and by using models such as the jigsaw approach, where students learn a portion of material individually, then teach it to peers, building both understanding and collaboration skills [31].

### Perceived strengths, opportunities and challenges in teaching medication adherence

Free-text answers were submitted by 208 respondents, expressing their thoughts on perceived strengths, opportunities and challenges in teaching medication adherence (Table 3). A frequently mentioned opportunity in teaching medication adherence was the encouragement of a better understanding of the topic amongst students and HPCs by providing a rigorous theoretical background, which can improve medication management and improving patient outcomes. Several respondents replied that challenges lie in creating a profound understanding of the multifaceted problem of non-adherence, thereby risking a paternalistic attitude amongst HCPs. Further, respondents mentioned that available methods for identifying and measuring adherence are insufficient, therefore the topic can be seen as theoretical and not applicable in real life settings.

**Table 3:** Identified themes from free-text answeres as well as subthemes

	Opportunities/Strengths	Challenges	Other thoughts
*Themes in medication adherence*	I. Strategies to monitor and support adherence to optimize medication management II. Deeper understanding of the impact of adherence on effectiveness of treatment III. Research can support teaching of theories and methods IV. Shared decision making can improve patient outcomes	I. Teaching impact and effectiveness of adherence measurements and interventions without obvious clinical signs of improvement II. Requires knowledge in health economics, anthropology, and behavioral science III. Lack of reliable measurement methods IV. Attitudes for shared decision making need to be developed amongst HCPs	I. Different effects of adherence to different therapies and in different adherence phases II. The economic aspect is often overlooked III. Consequence of national politics IV. Explore why adherence or concordance may not occur V. Support the development of practical skills
*Teaching strategies*	I. Involve research in teaching II. Use mixed teaching methods III. Raise awareness, move from patient compliance to patient concordance IV. Repeated in several courses, not taught as a stand-alone subject but integrated in different content	I. Practice medication adherence skills in clinical field practice, in small groups II. Theoretical and abstract topic for students III. Paternalistic attitude amongst students and HCPs	I. Covering the subject in several courses across the education is important II. Beneficial to start with theory, practical training and to involve students in research
*Teaching material*	I. Literature II. Innovative tools III. Research IV. Patient stories	I. Lack of standardized curricula II. A structured framework is requested	I. Create standardized curricula II. Guidelines on how to teach about the topic are requested
*Interprofessional collaboration*	I. Opportunity for interprofessional collaborations	I. Not taught interprofessionally	I. Extension to other disciplines is desirable II. Shared decision making with patients and other care givers
*MATech*	I. Current measurement methods are limited, potential solution with more accurate and real-time data	I. Not available during teaching or internship	I. Modernize how medication adherence is taught by including elements such as MATech
*Time*		I. Time consuming to coordinate interprofessional activities II. Overfilled curricula, and limited time to include more medication adherence III. Limited time to perform practical exams	I. Increase medication adherence content by giving it a bigger part of the syllabus and more time in the course

#### Teaching strategies

Responses on the opportunities in teaching medication adherence were centralised around the benefits of early introduction of the topic to students, and raising awareness by using mixed-method teaching. Methods menitoned by respondents were: theoretical teaching, laboratory practice, patient involvement, group work and patient simulation. Patient communication tools were mentioned frequently as something important to include in the teaching of medication adherence management. A frequently mentioned challenge was the lack of guidance on how to teach medication adherence, leading to inconsistencies in the depth and quality of education provided. Further, medication adherence is not taught as a stand alone topic, but integrated in the courses, often across several courses which was mentioned as a challenge by some respondents and as an opportunity by others.

#### Teaching materials

Respondents frequently stated that using research-based materials as well as available literature is important. Furthermore, several respondents emphasized the importance of patient involvement or using patient stories in the teaching for a broader understanding of the topic. The lack of guidelines was mentioned as a challenge in choosing teaching materials. Also, respondents expressed a lack of information on how to best approach student learning on adherence-related topics, also resulting in inconsistency in depth and quality of education.

#### Interprofessional collaboration

Interprofessional collaborations were mentioned as a prospect to optimize adherence monitoring and management and were described as an opportunity to create a team-based approach between HCPs by interprofessional learning and working. Several respondents expressed a willingness to increase interprofessional collaborations, however stating that it becomes a question of national politics in a real-life setting.

#### Medication adherence technologies

MATech was mentioned as an opportunity to modernize the topic by including technologies to a higher extent in teaching. Several respondents mentioned that MATech is a potential solution by delivering more reliable and real-time medication adherence data. However, there is a lacking availability of MATech both in teaching, internships and in practice.

#### Time

The time aspect of teaching was frequently mentioned as a challenge in teaching medication adherence. Respondents stated that it is a barrier that the course curriculum is already busy and that medication adherence is a complex topic requiring sufficient time to raise awareness and interest among students. Furthermore, respondents also expressed lack of time to perform practical exams.

### Curricula inventory

In total, 76 curricula were submitted by respondents from 24 different countries (see Additional file 2.). The curricula included in this study contained the course description and learning objectives, specific for each course and university. The submitted curricula covered courses such as clinical pharmacy, clinical pharmacology, medical pharmacology, social pharmacy, clinical internships, and general practice. In total 116 key words were identified.

**Fig. 5:**
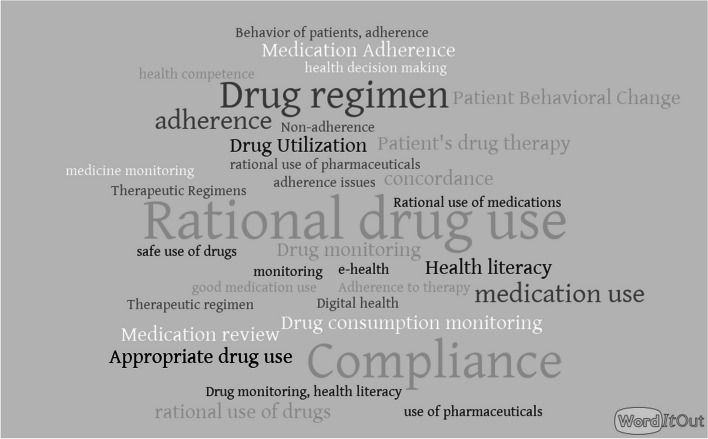
Identified key words in curricula of courses teaching medication adherence in European universities. Presented as a word cloud where the size of the words implicates the frequency of identification

The frequency of key words in submitted curricula was visualized using the word cloud [32]. One key word addressing the content of medication adherence was found in 21 submitted curricula (28%). Amongst reviewed curricula, one contained six key words while 24 (31%) curricula did not include any key word addressing medication adherence. The most common key word used were “rational drug use” followed by “medication adherence” and “compliance” (Fig. 5).

## Discussion

In this study, assessing to what extent medication adherence is covered by the pregraduate curricula for HPCs at European universities, we found that while medication adherence is recognized as an important topic, the content and depth of medication adherence teaching vary substantially. Today, the content of medication adherence in the curricula of courses is described mainly by using the key words “rational drug use”, “medication adherence” and “compliance”, although “compliance” is obsolete.

Respondents pursuing research in medication adherence rated the importance of increasing the teaching of medication adherence as highest. They have likely developed a deeper understanding of the topic and its implication on clinical, economic, and social outcomes. It also illustrates the importance of integrating research in the education of HCPs [33]. Respondents teaching nursing students rated the importance of medication adherence lowest, perhaps due to lesser engagement in research on the topic or familiar with pharmacology, compared to the other groups. However, research confirms that nurses have an important role in improving medication adherence, as do pharmacists and physicians [11, 15, 34, 35].

Additionally, a contributing factor to the limited implementation of medication adherence in clinical- and community settings can be the difficulties in accurately measuring adherence, thus not only affecting the integration of medication adherence in patient centred clinical- and community practice but also reducing interest in the topic by HCPs working primarily with patient care and pharmacotherapy. Overall, pharmacy education included medication adherence to the highest extent. Pharmacy education focuses on pharmaceuticals and their use, while the education of nurses and physicians is more focused on different diseases and how to diagnose and manage them. Pharmacy education in Europe has traditionally been focused on drug discovery and development [36]. The European Association of Clinical Pharmacology and Therapeutics’ Education Working Group has launched several initiatives to improve and harmonize European pharmacotherapy education that are promising [37]. Also, there has been a shift in recent years towards emphasizing the pharmacist’s role in clinical care including a patient-centred pharmacy practice to support patients in their medication use [38]. Although pharmacy schools are at the forefront of medication adherence education, this leadership is only beginning to be reflected in scientific literature and in European guidelines on pharmacists’ practices in monitoring medication adherence as part of interprofessional practice with physicians and nurses [39]. Further guidance might be available on local and national guidelines, which are difficult to assess because of language barriers [15].

Our results have important implications for pharmacists as a core profession in medication management. Patient-centred pharmacy practice can be utilized in the consultation process by implementing shared problem solving and shared decision making. Hence, supporting the patient in their medication use, reducing risk for adverse drug reactions and increasing medication adherence [40, 41]. Frameworks in the teaching patient-centred communication for undergraduate HCPs have previously been suggested in the literature [42, 43]. Thus, including medication adherence training may enhance patient-centred communication practice by equipping HCPs with tools for effective communication on medication use and shared-decision making.

Interprofessional collaboration can have a positive impact on both medication adherence and clinical outcomes of patients but was not implemented to a high extent in any of the three educations. Many respondents considered medication adherence as an opportunity to enhance collaboration, and to incorporate interprofessional teaching in the education of HCPs. Strong communication and well defined roles and responsibilities of HCPs have been identified as important factors to reach these benefits [10].

The clinical impact of non-adherence was the topic covered to the highest degree across all educations. The clinical outcomes associated with adherence are of significant importance to HCPs, given that it covers the effectiveness of treatment as a function of adherence, mortality or morbidity in relation to how patients follow their medication plan [44, 45]. Several respondents commented that medication adherence is not taught as a stand-alone subject and that it is incorporated in teaching across several courses, also expressing that the time spent on the topic is sparse. Hence, the clinical outcomes have likely been incorporated where relevant, as a part of a therapeutic area. While the clinical impact of non-adherence is essential knowledge, it is equally important to address preventive strategies that equip HCPs with actionable tools. These include methods for identifying and measuring non-adherence, as well as approaches for monitoring and supporting medication adherence.

Preventive measures were addressed to varying extents, with patient-reported methods for identifying and measuring adherence being the most extensively covered across educational programs. Patient education appeared as the most frequently taught approach for monitoring and supporting adherence, reflecting clinical practice where patient-reported methods are commonly applied for adherence assessment. However, research has shown that this method often overestimates patient adherence [46]. Medication management, which includes review of medication list, management of missed doses, and management of side effects and interactions, was the second most taught measure for monitoring and supporting medication adherence. Medication management and patient education are important topics in medication adherence, focusing on empowering the patient by using shared-decision making, considering patients’ needs, simplifying, structuring and identifying the right, individual tools to follow a medication plan [46–48]. Still, HCPs have expressed a need to improve competence in monitoring and evaluation methods, and shared-decision making methods in order to engage patients in taking an active role in medication adherence management [12, 18].

Digital technologies have a positive effect on medication management, shared-decision making and overall improve the quality of care [49]. The implementation of these tools in clinical practice is still rare [15, 18, 50], which is reflected in the results of this study, reporting a relatively low inclusion of MATech in teaching. Yet, several respondents expressed a positive attitude towards teaching about digital tools. This calls for further initiatives to inform and educate university teachers about these technologies.

Lectures were reported as the predominantly used method of teaching medication adherence while respondents stated that practical training, and mixed-method learning are more beneficial methods. This is in line with the general shift in the societal view of medical education toward instruction methods that enables active and student-centred learning compared to traditional lectures where knowledge is passively provided by the lecturer to students [51]. However, it seems to take time for this transition to occur in practice and more efforts are thus needed to stimulate other teaching methods. Medication adherence and interprofessional collaboration ought to be a suitable topic for such activities by e.g. the use of student-run medication review and patient education teams in outpatient clinics [52, 53].

Several respondents expressed a lack of information on how to teach medication adherence. Many of them requested guidelines or standardized curricula to ensure a common level of quality and depth in how the topic is taught. However, respondents also expressed that teaching on medication adherence does not yet reflect the reality of what and how it is implemented in practice, but rather what and how it should be. A recent study found that across Europe, the most significant hurdles in medication adherence management were lack of time, availability of digital solutions and awareness amongst patients [18]. Hence, there is a request to not only standardize the teaching of medication adherence, but also to use teaching as an effective strategy to translate and implement knowledge and skills of future HCPs into practice.

### Strengths & weaknesses

The main strength of this study is the pan-European approach with responses from 34 European countries and 142 universities. The survey was constructed by an international research team, with in-depth expertise of the topic and the survey was subjected to pilot study for refinement and reliability testing. Furthermore, this study applies triangulation of results, combining review of curricula, thematic analysis of free-text replies and analysis of quantitative survey data. The spread of data analysis adds validity and credibility to the results [54, 55]. All qualitative results were analyzed using a peer-reviewed approach, adding validation of the conclusions drawn from qualitative data results [54]. Finally, the survey was conducted in three different languages to ensure broad participation.

This study also faced some limitations, primarily due to the recruitment of respondents, which mainly successful by personal invitations from the research team and by contacts through ENABLE COST Action members, which cannot rule out selection bias. The response rates from different countries also varied greatly, which may have impacted on our results. Thus, we did not consider it feasible to make any comparisons between countries. Lastly, there may be responder bias, as individuals with a particular interest in medication adherence could be more inclined to participate in the survey.

## Conclusion

Medication adherence teaching varies across European educational programs in medicine, pharmacy and nursing. Most teachers consider the topic of medication adherence important in the education of future healthcare professionals. However, insufficient hours are dedicated to this topic, and efforts should be made to facilitate the teaching of effective methods to monitor and support patients to improve medication adherence. Standardizing curricula and teaching methods is needed to increase competence, promote interprofessional collaboration and integrate medication adherence management in everyday practice.

## Supplementary Information

Additional file 1.

Additional file 2.

## Data Availability

The data sets used and analyzed during the current study are available from the corresponding author on reasonable request.

## Abbreviations

MA: Medication adherence
HCP: Health care professional
MATech: Medication adherence Technology
CI95%: 95% Confidence interval
e.g.: For example
i.e.: In other words

## References

[1] Vrijens B, De Geest S, Hughes DA, Przemyslaw K, Demonceau J, Ruppar T, et al. A new taxonomy for describing and defining adherence to medications. Br J Clin Pharmacol. 2012;73(5):691–705. 22486599 10.1111/j.1365-2125.2012.04167.x PMC3403197

[2] Adherence to long-term therapies: evidence for action. Geneva, Switzerland: World Health Organization; 2003. Available from: https://iris.who.int/handle/10665/42682

[3] Imagine tomorrow: report on the 2nd WHO global forum on innovation for ageing populations, Kobe, Japan, 7–9 October 2015. Kobe, Japan: WHO Centre for Health Development; 2016 p. 91 p. Available from: https://iris.who.int/handle/10665/205288

[4] Kardas P, Lewek P, Matyjaszczyk M. Determinants of patient adherence: a review of systematic reviews. Front Pharmacol. 2013:4(91). 10.3389/fphar.2013.00091. 10.3389/fphar.2013.00091 PMC3722478 23898295

[5] Conn VS, Ruppar TM, Enriquez M, Cooper P. Medication adherence interventions that target subjects with adherence problems: Systematic review and meta-analysis. Res Soc Adm Pharm. 2016;12(2):218–46. 10.1016/j.sapharm.2015.06.001 PMC4679728 26164400

[6] Brinkman DJ, Tichelaar J, Mokkink LB, Christiaens T, Likic R, Maciulaitis R, et al. Key Learning Outcomes for Clinical Pharmacology and Therapeutics Education in Europe: A Modified Delphi Study. Clin Pharmacol Ther. 2018;104(2):317–25. 29205299 10.1002/cpt.962 PMC6099198

[7] Costa E, Pecorelli S, Giardini A, Savin M, Menditto E, Lehane E, et al. Interventional tools to improve medication adherence: review of literature. Patient Prefer Adherence. 2015;9:1303–14. 26396502 10.2147/PPA.S87551 PMC4576894

[8] Nieuwlaat R, Wilczynski N, Navarro T, Hobson N, Jeffery R, Keepanasseril A, et al. Interventions for enhancing medication adherence. Cochrane Database Syst Rev. 2014:11.CD000011. 10.1002/14651858.CD000011.pub4. 10.1002/14651858.CD000011.pub4 PMC7263418 25412402

[9] Ha JF, Longnecker N. Doctor-patient communication: a review. Ochsner J. 2010;10(1):38–43. 21603354 PMC3096184

[10] Celio J, Ninane F, Bugnon O, Schneider MP. Pharmacist-nurse collaborations in medication adherence-enhancing interventions: A review. Patient Educ Couns. 2018;101(7):1175–92. 29628282 10.1016/j.pec.2018.01.022

[11] Zoromski LM, Frazier S. Nurses’ role in promoting medication adherence. Nursing (Lond). 2023;53(1):39–44. 10.1097/01.NURSE.0000902956.76232.93 36573869

[12] Kvarnström K, Airaksinen M, Liira H. Barriers and facilitators to medication adherence: a qualitative study with general practitioners. BMJ Open. 2018;8(1):e015332. 10.1136/bmjopen-2016-015332. 10.1136/bmjopen-2016-015332 PMC5786122 29362241

[13] Rathbone AP, Mansoor SM, Krass I, Hamrosi K, Aslani P. Qualitative study to conceptualise a model of interprofessional collaboration between pharmacists and general practitioners to support patients’ adherence to medication. BMJ Open. 2016;6(3):e010488. 10.1136/bmjopen-2015-010488. 10.1136/bmjopen-2015-010488 PMC4800113 26983948

[14] Clyne W, McLachlan S, Mshelia C, Jones P, De Geest S, Ruppar T, et al. My patients are better than yours: ‘optimistic bias about patients’ medication adherence by European health care professionals. Patient Prefer Adherence. 2016;2016(10):1937–44. 10.2147/PPA.S108827 PMC5045226 27713621

[15] Clyne W, Mshelia C, McLachlan S, Jones P, De Geest S, Ruppar T, et al. A multinational cross-sectional survey of the management of patient medication adherence by European healthcare professionals. BMJ Open. 2016;6(2):e009610. 10.1136/bmjopen-2015-009610. 10.1136/bmjopen-2015-009610 PMC4746470 26832430

[16] Schneider MP, Aslani P. Adherence policy, education and practice: an international perspective. Pharm Pract Internet. 2010;8(4):209–12. 10.4321/s1886-36552010000400001 PMC4127057 25126142

[17] White S, Clyne W, Mshelia C. An educational framework for managing andsupporting medication adherence in Europe. Pharm Educ. 2013;13(1):118–20.

[18] Hafez G, Aarnio E, Mucherino S, Kamusheva M, Qvarnström M, Potočnjak I, et al. Barriers and Unmet Educational Needs Regarding Implementation of Medication Adherence Management Across Europe: Insights from COST Action ENABLE. J Gen Intern Med. 2024;39:2917–26. 38941058 10.1007/s11606-024-08851-2 PMC11576669

[19] Van Boven JF, Tsiligianni I, Potočnjak I, Mihajlović J, Dima AL, Nabergoj Makovec U, et al. European network to advance best practices and technology on medication adherence: mission statement. Front Pharmacol. 2021;12:748702. 10.3389/fphar.2021.748702. 34707502 10.3389/fphar.2021.748702 PMC8544003

[20] Eltaybani S, Igarashi A, Yamamoto-Mitani N. Palliative and end-of-life care education in prelicensure nursing curricula: a nationwide survey in an Arab country. Nurse Educ Today. 2021;96:104644. 10.1016/j.nedt.2020.104644. 33242705 10.1016/j.nedt.2020.104644

[21] Svensberg K, Björnsdottir I, Wallman A, Sporrong SK. Nordic pharmacy schools’ experience in communication skills training. Am J Pharm Educ. 2017;81(9):6005. 10.5688/ajpe6005. 29302085 10.5688/ajpe6005 PMC5738943

[22] Kardas P, Dima AL, Potočnjak I, Wettermark B, Agh T. Editorial: Recent advances in attempts to improve medication adherence-from basic research to clinical practice. Front Pharmacol. 2023;14:1144662. 10.3389/fphar.2023.1144662. 36843921 10.3389/fphar.2023.1144662 PMC9946449

[23] Bandiera C, Ribaut J, Dima AL, Allemann SS, Molesworth K, Kalumiya K, et al. Swiss priority setting on implementing medication adherence interventions as part of the European ENABLE COST Action. Int J Public Health. 2022;67:1605204. 10.3389/ijph.2022.1605204. 36032275 10.3389/ijph.2022.1605204 PMC9411421

[24] Eccles DW, Arsal G. The think aloud method: what is it and how do I use it? Qual Res Sport Exerc Health. 2017;9(4):514–31. 10.1080/2159676X.2017.1331501.

[25] Nabergoj Makovec U, Goetzinger C, Ribaut J, Barnestein-Fonseca P, Haupenthal F, Herdeiro MT, et al. Developing a medication adherence technologies repository: proposed structure and protocol for an online real-time Delphi study. BMJ Open. 2022:12(4):e059674. 10.1136/bmjopen-2021-059674. 10.1136/bmjopen-2021-059674 PMC9074304 35459677

[26] Valdez D, Montenegro MS, Crawford BL, Turner RC, Lo WJ, Jozkowski KN. Translation frameworks and questionnaire design approaches as a component of health research and practice: a discussion and taxonomy of popular translation frameworks and questionnaire design approaches. Soc Sci Med. 2021;278:113931. 10.1016/j.socscimed.2021.113931. 33905986 10.1016/j.socscimed.2021.113931

[27] Harris PA, Taylor R, Minor BL, Elliott V, Fernandez M, O’Neal L, et al. The REDCap consortium: building an international community of software platform partners. J Biomed Inform. 2019;95:103208. 10.1016/j.jbi.2019.103208. 31078660 10.1016/j.jbi.2019.103208 PMC7254481

[28] Harris PA, Taylor R, Thielke R, Payne J, Gonzalez N, Conde JG. Research electronic data capture (REDCap) - A metadata-driven methodology and workflow process for providing translational research informatics support. J Biomed Inform. 2009;42(2):377–81. 18929686 10.1016/j.jbi.2008.08.010 PMC2700030

[29] Maguire M, Delahunt B. Doing a Thematic Analysis: A Practical, Step-by-Step Guide for Learning and Teaching Scholars. AISHE-J Irel J Teach Learn High Educ. 2017;9(3):3351–33514.

[30] Fife ST, Gossner JD. Deductive Qualitative Analysis: Evaluating, Expanding, and Refining Theory. Int J Qual Methods. 2024:23.

[31] Cochon Drouet O, Lentillon-Kaestner V, Margas N. Effects of the Jigsaw method on student educational outcomes: systematic review and meta-analyses. Front Psychol. 2023;14:1216437. 10.3389/fpsyg.2023.1216437. 37599768 10.3389/fpsyg.2023.1216437 PMC10436097

[32] DePaolo CA, Wilkinson K. Get your head into the clouds: using word clouds for analyzing qualitative assessment data. TechTrends. 2014;58(3):38–44. 10.1007/s11528-014-0750-9.

[33] Ahmed Y, Taha M, Khayal S. Integrating Research and Teaching in Medical Education: Challenges, Strategies, and Implications for Healthcare. J Adv Med Educ Prof. 2024:12(1). 10.30476/JAMP.2023.99751.1854 PMC10837463 38313425

[34] Van Camp YP, Van Rompaey B, Elseviers MM. Nurse-led interventions to enhance adherence to chronic medication: systematic review and meta-analysis of randomised controlled trials. Eur J Clin Pharmacol. 2013;69(4):761–70. 10.1007/s00228-012-1419-y. 23052418 10.1007/s00228-012-1419-y

[35] De Baetselier E, Dilles T, Feyen H, Haegdorens F, Mortelmans L, Van Rompaey B. Nurses’ responsibilities and tasks in pharmaceutical care: A scoping review. Nurs Open. 2022;9(6):2562–71. 34268910 10.1002/nop2.984 PMC9584497

[36] Atkinson J, Rombaut B. The 2011 PHARMINE report on pharmacy and pharmacy education in the European Union. Pharm Pract Internet. 2011;9(4):169–87. 10.4321/s1886-36552011000400001 PMC3818732 24198854

[37] Bakkum MJ, Donker EM, Spitaleri Timpone P, Hagen CAM, Richir MC, Van Agtmael MA, et al. Educational value of international and intercultural differences in prescribing: the international and interprofessional student-run clinic project. Eur J Clin Pharmacol. 2023;79(4):571–4. 36749353 10.1007/s00228-023-03465-9 PMC10038944

[38] Atkinson J. Heterogeneity of Pharmacy Education in Europe. Pharmacy. 2014;2(3):231–43.

[39] Therapeutic patient education: an introductory guide. Copenhagen: WHO Regional Office for Europe; 2023. Available from: https://www.who.int/europe/publications/i/item/9789289060219

[40] Wolters M, Van Hulten R, Blom L, Bouvy ML. Exploring the concept of patient centred communication for the pharmacy practice. Int J Clin Pharm. 2017;39(6):1145–56. 28887610 10.1007/s11096-017-0508-5 PMC5694524

[41] Ilardo ML, Speciale A. The Community Pharmacist: Perceived Barriers and Patient-Centered Care Communication. Int J Environ Res Public Health. 2020;17(2):536. 31952127 10.3390/ijerph17020536 PMC7013626

[42] Von Fragstein M, Silverman J, Cushing A, Quilligan S, Salisbury H, Wiskin C, et al. UK consensus statement on the content of communication curricula in undergraduate medical education. Med Educ. 2008;42(11):1100–7. 18761615 10.1111/j.1365-2923.2008.03137.x

[43] Wolters M, Van Paassen JG, Minjon L, Hempenius M, Blokzijl MR, Blom L. Design of a Pharmacy Curriculum on Patient Centered Communication Skills. Pharmacy. 2021;9(1):22. 33467691 10.3390/pharmacy9010022 PMC7838998

[44] Ho PM, Magid DJ, Shetterly SM, Olson KL, Maddox TM, Peterson PN, et al. Medication nonadherence is associated with a broad range of adverse outcomes in patients with coronary artery disease. Am Heart J. 2008;155(4):772–9. 18371492 10.1016/j.ahj.2007.12.011

[45] Rasmussen JN, Chong A, Alter DA. Relationship Between Adherence to Evidence-Based Pharmacotherapy and Long-term Mortality After Acute Myocardial Infarction. JAMA. 2007Jan 10;297(2):177. 17213401 10.1001/jama.297.2.177

[46] Krueger KP, Berger BA, Felkey B. Medication adherence and persistence: A comprehensive review. Adv Ther. 2005;22(4):313–56. 16418141 10.1007/BF02850081

[47] Brown MT, Bussell J, Dutta S, Davis K, Strong S, Mathew S. Medication Adherence: Truth and Consequences. Am J Med Sci. 2016;351(4):387–99. 27079345 10.1016/j.amjms.2016.01.010

[48] Jimmy B, Jose J. Patient Medication Adherence: Measures in Daily Practice. Oman Med J. 2011;26(3):155–9. 22043406 10.5001/omj.2011.38 PMC3191684

[49] Pouls BPH, Vriezekolk JE, Bekker CL, Linn AJ, Van Onzenoort HAW, Vervloet M, et al. Effect of Interactive eHealth Interventions on Improving Medication Adherence in Adults With Long-Term Medication: Systematic Review. J Med Internet Res. 2021:23(1). 10.2196/18901 PMC7822716 33416501

[50] Mason M, Cho Y, Rayo J, Gong Y, Harris M, Jiang Y. Technologies for medication adherence monitoring and technology assessment criteria: narrative review. JMIR MHealth UHealth. 2022;10(3):e35157. 10.2196/35157. 35266873 10.2196/35157 PMC8949687

[51] Mehta NB, Hull AL, Young JB, Stoller JK. Just imagine: new paradigms for medical education. Acad Med. 2013;88(10):1418–23. 10.1097/ACM.0b013e3182a36a07. 23969368 10.1097/ACM.0b013e3182a36a07

[52] Reumerman MO, Richir MC, Domela Nieuwenhuis PM, Sultan R, Daelmans HEM, Springer H, et al. The clinical and educational outcomes of an inter-professional student-led medication review team, a pilot study. Eur J Clin Pharmacol. 2021;77(1):117–23. 32770387 10.1007/s00228-020-02972-3 PMC7782385

[53] Sultan R, Van Den Beukel TO, Reumerman MO, Daelmans HEM, Springer H, Grijmans E, et al. An Interprofessional Student-Run Medication Review Program: The Clinical STOPP/START-Based Outcomes of a Controlled Clinical Trial in a Geriatric Outpatient Clinic. Clin Pharmacol Ther. 2022;111(4):931–8. 34729774 10.1002/cpt.2475 PMC9299053

[54] Johnson JL, Adkins D, Chauvin S. A Review of the Quality Indicators of Rigor in Qualitative Research. Am J Pharm Educ. 2020;84(1):7120. 32292186 10.5688/ajpe7120 PMC7055404

[55] Bowen GA. Document Analysis as a Qualitative Research Method. Qual Res J. 2009;9(2):27–40.

