# Supplementary material for: Medication adherence in the curricula of future European physicians, pharmacists and nurses – a cross-sectional survey
Source: BMC Med Educ. 2025 Mar 5;25:339. doi: 10.1186/s12909-025-06909-1 (PMC11881433; doi:10.1186/s12909-025-06909-1)
Supplement: Supplementary file 1 — Additional file 1. [file 12909_2025_6909_MOESM1_ESM.pdf]

Questionnaire: Medication adherence in the curricula of pharmacy-, medical- and nursing students

Medication non-adherence can result in increased morbidity, mortality, and healthcare costs. To improve medication non-adherence, pharmacists, doctors, and nurses play a crucial role. However, there is currently no clarity on what must be taught in the education programs regarding medication adherence. To shed light on this issue, this study will map how, and which parts of medication adherence are implemented in the curricula of future European pharmacists, doctors, and nurses. Specifically, course leaders and teachers who are involved in courses or have knowledge about courses which include medication adherence are the respondents of this questionnaire.

The study is undertaken through a collaborative effort between Uppsala University and the EU COST Enable initiative project on digital tools and adherence. For comprehensive details on this project, ethical considerations, as well as information regarding the handling and storage of data, see the attached file below.

Information letter attachment

It takes approximately 10 minutes to fill in the questionnaire.

Contact information:

Hanna Gottlieb, Department of Pharmacy, Uppsala University, Uppsala, Sweden.

hanna.gottlieb@farmaci.uu.se

<u>Section one - Respondent characteristics</u>

The first section consists of sociodemographic questions.

1. **Gender**
- Male
- Female
- Other/ prefer not to say

2. **What is your role within the university department/research institute?**
- Course leader
- Teacher of a course
- Teacher not involved in the course
- Other*
- Prefer not to say

*Describe your role within the university department/research institute

3. **How many years of experience do you have in teaching of medical doctors, nurses and pharmacists?**
- No previous experience
- 1-5 years
- 6-10 years
- 11-15 years
- 16-20 years
- More than 20 years
- Prefer not to say

4. **What is your highest academic degree?**
   i.e. Bachelor, Master, PhD, Assoc. Prof, Prof

5. **Do you do research on medication adherence?**
   - Not now, but previously
   - Yes, I do
   - No Prefer not to say

6. **What do you think is the best way to teach about medication adherence?**
   You can tick maximum 3 boxes.

   - Lectures
   - Practice laboratory
   - Clinical field practice
   - Group discussion
   - Assigned reading
   - Writing assignment
   - Other*
   - Don’t know

   *Describe other way to teach about medication adherence.

7. Estimate the total hours of medication adherence in pre-graduate teaching of medical doctors at your university.
An estimated value is enough.

- 1 - 2 hours
- ½ - 1 day
- 2 - 5 day
- Other*
- Don't know

*Provide total hours of medication adherence in pre-graduate teaching of medical doctors at your university.

8. Estimate the total hours of medication adherence in pre-graduate teaching of nurses at your university.
An estimated value is enough.

- 1 - 2 hours
- ½ - 1 day
- 2 - 5 day
- Other*
- Don't know

*Provide total hours of medication adherence in pre-graduate teaching of nurses at your university.

9. Estimate the total hours of medication adherence in pre-graduate teaching of pharmacists at your university.
An estimated value is enough.

- 1 - 2 hours
- ½ - 1 day
- 2 - 5 day
- Other*
- Don't know

*Provide total hours of medication adherence in pre-graduate teaching of pharmacists at your university.

10. Do you think that there should be paid more attention to medication adherence during the medical doctor-/pharmacy- /nursing education?

(Strongly disagree) 0-1-2-3-4-5-6-7-8-9-10 (Strongly agree)

11. To your knowledge, what course(s) related to medication adherence are there in your university, faculty or institute?

12. In which country is/are this/these course(s) conducted in?

13. In which university is/are this/these course(s) conducted in?

<u>Section two - Characteristics of the course(s)</u>

Now three sections follow, each section consists of specific course characteristics.

14. **What is the name of the course(s) related to medication adherence that you teach, or you are involved in?**
    Please list all courses in the notes box.

15. **Please provide the link to the course description/syllabus of the course(s).**
    Please provide link for all of the courses in the notes box

16. **Are the course(s) for pharmacy-, medical- and/or nursing students?**
    You can tick more than one box.

    - Medical students
    - Nursing students
    - Pharmacy students

17. **Who are the main targets of the course(s)?**
    You can tick more than one box.

    - Bachelor students
    - Master students
    - Other*
    - Don't know

    *Describe other main target of the course(s).

18. **Is any of the course(s) interdisciplinary between medical-/pharmacy-/nursing school and other professions? If so, which students from other educations take part?**
    i.e., physiotherapy- or public health students.

    - Yes *
    - No
    - Don't know

    * List other educations that take part in the course(s).

19. **Are any patients involved in the course(s)?**

    - Yes
    - No
    - Don't know

20. **Which teaching materials are used for medication adherence in the course(s)?**
You can tick more than one box.

- Scientific articles
- Teaching books
- National or international guidelines
- Scientific organizations: ESPACOMP, ENABLE, Skills4Adherence etc.
- Own research
- Other*
- Don't know

* Describe other teaching materials used in the course(s).

Are the following topics covered in the course(s) you are teaching/responsible for?
Please check the correct boxes. You can tick more than one box.

Domain 1: Definitions and terms

Domain 2: Identifying and measurement

Domain 3: Monitoring and supporting

Domain 4: Consequences and outcomes

21. **Domain 1: Which of the definitions and terms are covered in the course(s)?** ¨
You can tick more than one box.

- **The definition of adherence:** "The extent to which a person's behavior - taking medication, following a diet, and/or executing lifestyle changes, corresponds with agreed recommendations from a health care provider".
- **The definition of adherence to medication:** "The process by which patients take their medications as prescribed, composed of initiation, implementation and discontinuation. Initiation occurs when the patient takes the first dose of a prescribed medication. Discontinuation occurs when the patient stops taking the prescribed medication, for whatever reason(s). Implementation is the extent to which a patient's actual dosing corresponds to the prescribed dosing regimen, from initiation until the last dose. Persistence is the length of time between initiation and the last dose, which immediately precedes discontinuation."
- **The definition of management of adherence:** "The process of monitoring and supporting patients' adherence to medications by health care systems, providers, patients, and their social networks."
- **Adherence in relation to compliance:** The degree to which a patient's behavior corresponds to the prescriber's advice.
- **Adherence in relation to concordance:** Equally involvement of the patient and health care professional in medication decision-making.
- **The five determinants of adherence:** Patient-related factors, health system related factors, socioeconomic factors, therapy related factors and condition related factors.
- **None of them**

22. **Domain 2: Which of the identifying and measurement methods are covered in the course(s)?**
    You can tick more than one box.

- Patient reported: Talking with the patients, interviewing them, having a survey.
- Manual pill count and electronic detection of package entry.
- Direct measurements: drug or drug metabolite monitoring.
- Registries: Electronic prescription data, refill/dispensing data form pharmacies and electronic health records (EHRs)
- Another method to measure medication adherence ___________
- None of them

23. **Domain 3: Which of the monitoring and supporting medication adherence methods are covered in the course(s)?**
    You can tick more than one box.

    - **Medication Management:** Medication regimen management/ medication review/ Management of missed doses/ A pill card/ Management of side effects and interactions
    - **Interprofessional collaboration and communication:** Integrated care interventions
    - **Patient provider communication:** Listening/ Communicating/ Context
    - **Patient Education:** Sharing information/ Shared decision-making/ Understanding/ Exploring options
    - **Patient engagement:** Memory enhancement/ Reminders/ Self-monitoring training and feedback/ Self-management
    - **Behavioral change techniques:** Emotional-based change techniques/ Action-based behavioral change techniques
    - **Social Factors:** Peers/ Social support/ In group
    - **Digital tools:** Remotely/ Electronic drug monitoring/ Electronic medication managements systems
    - **Another method to monitor or to support medication adherence____________**
    - **None of them**

24. **Domain 4: Which of the consequences and outcomes of medication non-adherence are covered in the course(s)?**
    You can tick more than one box.

- Economic impacts
- Clinical impacts
- Social impacts
- None of them

25. Which teaching strategies are used for each domain?
You can tick more than one box.

- Lecture
- Practice laboratory
- Clinical field practice
- Group discussion
- Assigned reading
- Writing assignment
- Self-study
- Case studies
- Another method: ___________________
- Don't know

26. Which form(s) of examinations is used, related to the teaching of medication adherence in the course(s)?
You can tick more than one box.

- Written exam
- Oral exam
- Essay
- A practical exam
- Other:
- Don't know

27. What do you think are strengths/opportunities related to the teaching of medication adherence in the course(s)?

28. What do you think are the challenges related to the teaching of medication adherence in the course(s)?

29. Do you teach about Medication Adherence Technologies (MATech) in the course(s)?
i.e. digital inhalers, smartphone applications, electronic auto-injector devices or bioingestible sensors.

- Yes*
- No
- Don't know

* What type of Medication Adherence Technologies are teached in the course(s)?

30. Do you have any additional thoughts on medication adherence and its teaching?

Thank you for taking the survey.

Have a nice day!
