# Supplementary material for: Medication adherence in the curricula of future European physicians, pharmacists and nurses – a cross-sectional survey
Source: BMC Med Educ. 2025 Mar 5;25:339. doi: 10.1186/s12909-025-06909-1 (PMC11881433; doi:10.1186/s12909-025-06909-1)
Supplement: Supplementary file 2 — Additional file 2. [file 12909_2025_6909_MOESM2_ESM.pdf]

Universities participating in questionnaire

Country	*University*
Albania	University of Elbasan "Aleksandër Xhuvani"
	University of Vlore Ismail Qemali
	Albania University
Belgium	KU Leuven
	University Libre de Bruxelles
	Vrije University of Brussel
	University of Antwerp
Bosnia and Herzegovina	University of East Sarajevo
	University of Sarajevo
Bulgaria	Medical University of Sofia
Croatia	University of Zagreb
	University of Osijek
	University of Applied Health Sciences
	University of Rijeka
Czech Republic	Charles University
	South Bohemian University
Denmark	University of Copenhagen
	VIA University College
Estonia	University of Tartu
Finland	Metropolia University of Applied Sciences
	Åland University of Applied Sciences
France	University Grenoble Alpes
	Paul Sabatier University
	University of Angers
	Paris-Saclay University
	University of Toulouse
	University of Strasbourg
	University of Bordeaux
	Lyon East Faculty of Medicine
	The Institut de Formation en Soins Infirmiers (IFSI) du Vinatier in Lyon
	Grenoble Alpes University
	University in Nantes
	Claude Bernard University Lyon 1
	University of Tours
	Grenoble Alpes University
	University of Rouen Normandy
Germany	University of Bonn
	University of Greifswald
	Kiel University
	The Ludwig Maximilian University of Munich
Greece	University of Crete

Hungary	University of Pécs
	University of Szeged
Iceland	University of Iceland
Ireland	University College Cork
	University of Medicine and Health Sciences
Italy	University of L'Aquila
	University of Bologna
	University of Eastern Piedmont
	University of Milan
	University of Verona
	University of Salerno
Latvia	Riga Stradins University
	University of Latvia
Lithuania	Vilnius University
Malta	University of Malta
Moldova	Nicolae Testemițanu State University of Medicine and Pharmacy
Netherlands	University of Groningen
North Macedonia	The Goce Delčev University of Štip
Norway	Oslo Metropolitan Unitersity
Poland	Medical University of Lodz
Portugal	University of Porto
	Polytechnic Institute of Guarda
	University of Minho
	University of Beira Interior
	NOVA University of Lisbon
	Lusófona University
Romania	University of Medicine and Pharmacy of Craiova
	Carol Davila University of Medicine and Pharmacy
	Grigore T. Popa University of Medicine and Pharmacy
	University of Medicine and Pharmacy Iuliu Hatieganu Cluj-Napoca
	Victor Babes University of Medicine and Pharmacy Timisoara
Serbia	University of Novi Sad
	University of Belgrade
	University of Kragujevac
Slovakia	Comenius University
Slovenia	University of Ljubljana
Spain	University of Zaragoza
	University of Málaga
	Rey Juan Carlos University
	The Technical University of Madrid
	University of the Basque Country
Sweden	Uppsala University
	Gothenburg University
	Umeå University

	Karolinska Institute
	Linnaeus University
	Örebro University
	Lund University
	University of Gävle
	University of Borås
	Marie Cederschiöld University
	Kristianstad University
Switzerland	University of Basel
	Université de Genève
	University of Lausanne
	Haute École de Santé Vaud (HESAV)
Turkey	İstinye University
	Altinbas University
	Ataturk University
	Dokuz Eylul University
	Kırıkkale University
	Tekirdag Namik Kemal University
	Ankara University
	Hacettepe University
	Marmara University
Ukraine	Danyli Halytsky Lviv National Medical University
United Kingdom	University of Strathclyde
	University of Chichester
	University of East Anglia
	University of Portsmouth
	University of Greenwich
	York St John University
	Bangor University
	University of Brighton

Universities participating in curricula inventory

Country	*University*
Albania	University of Vlore Ismail Qemali
Belgium	KU Leuven
	University Libre de Bruxelles
	Vrije University of Brussel
Croatia	University of Zagreb
Czech Republic	Charles University
Denmark	University of Copenhagen
Estonia	University of Tartu
Finland	Åland University of Applied Sciences
Hungary	University of Pécs
Iceland	University of Iceland
Ireland	University College Cork
Italy	University of Bologna
Netherlands	University of Groningen
Norway	Oslo Metropolitan Unitersity
Portugal	University of Porto
	NOVA University of Lisbon
Romania	University of Medicine and Pharmacy of Craiova
	University of Medicine and Pharmacy Iuliu Hatieganu Cluj-Napoca
	Victor Babes University of Medicine and Pharmacy Timisoara
Serbia	University of Novi Sad
Spain	University of the Basque Country
Sweden	Uppsala University
	Umeå University
	Linnaeus University
	Örebro University
	Lund University
	University of Borås
Turkey	İstinye University
	Altinbas University
	Hacettepe University
	Marmara University
United Kingdom	University of East Anglia
	University of Greenwich
